# A Review of the Relationship Between Obesity and Immunotherapy Response in Cancer

**DOI:** 10.3390/ijms27198587

**Published:** 2026-09-25

**Authors:** Qiongyu Shi, Pedro Ramos, Carol Plazas Asaad, Tomas Alejandro Leibkuchler, Ashley R. Gaffey, Weizhou Zhang, Ryan Kolb

**Affiliations:** 1 Department of Pathology, Immunology and Laboratory Medicine, College of Medicine, University of Florida, Gainesville, FL 32610, USA; qshi1@ufl.edu (Q.S.); pedroramos@ufl.edu (P.R.); cplazasasaad@ufl.edu (C.P.A.); t.leibkuchler@ufl.edu (T.A.L.); ashley.gaffey@ufl.edu (A.R.G.); zhangw@ufl.edu (W.Z.); 2 Biomedical Sciences Graduate Program, College of Medicine, University of Florida, Gainesville, FL 32610, USA; 3 UF Health Cancer Institute, University of Florida, Gainesville, FL 32610, USA

**Keywords:** cancer, immunotherapy, obesity

## Abstract

Obesity is a growing public health concern, being correlated with several comorbidities including heart disease, liver disease, diabetes, and several cancers. It has been demonstrated that obesity increases the risk of developing several types of cancers as well as being associated with a worse prognosis, increased metastasis, increased recurrence, and worse response to therapy. Paradoxically, early clinical trials of immune checkpoint inhibitors found a correlation between obesity and increased response to immune checkpoint inhibitors—a phenomenon referred to as the obesity paradox. Since then, numerous studies have been done to further examine the correlation between obesity and response to immune checkpoint inhibitors, with both confirmatory and non-confirmatory results depending on the type of cancer. Many of these studies have found contradicting results, indicating a need for further studies and meta-analyses to validate or refute this correlation. Here, we review recent studies on the correlation between obesity and response to immune checkpoint inhibitors across different types of cancer, with an emphasis on meta-analyses when available, discuss hypothesized mechanisms for this correlation, and highlight the knowledge gaps that need to be filled.

## 1. Introduction

Obesity is a complex metabolic disorder defined by excessive accumulation of adipose tissue that poses significant risks to human health. The World Health Organization (WHO) defines overweight as a body mass index (BMI) of 25.0–29.9 kg/m^2^ and obesity as a BMI of 30 kg/m^2^ or greater, with further sub-classifications into Class I (BMI 30–34.9), Class II (BMI 35–39.9), and Class III (BMI ≥ 40) [1,2]. Over the past several decades, the number of individuals living with obesity has dramatically increased across all ages globally. In the United States, recent data from the National Health and Nutrition Examination Survey (NHANES) 2021–2023 reported that the overall prevalence of adult obesity was 40.3%, with severe obesity affecting 9.7% of adults [3]. Obesity is a well-established risk factor for numerous chronic diseases, including cardiovascular diseases, type 2 diabetes mellitus (T2DM), musculoskeletal disorders, non-alcoholic fatty liver disease (NAFLD), and multiple types of cancer [2,4,5,6].

Large-scale population studies have demonstrated that obesity is associated with increased risk for many cancer types, including breast, colorectal, pancreatic, liver, endometrial, renal, esophageal, etc. [4,7]. Emerging evidence suggests that obesity promotes tumor development and progression through a complex interplay of systemic metabolic dysregulation and changes in the tumor microenvironment (TME). For instance, obesity drives chronic low-grade inflammation with increased production of pro-inflammatory cytokines and adipokines, including tumor necrosis factor alpha (TNF-α), interleukin-6 (IL-6), interleukin-1 beta (IL-1β), and leptin, which promote tumorigenesis and support tumor cell survival [8,9,10]. Obesity can also promote insulin resistance and hyperinsulinemia, leading to elevated circulating levels of insulin and insulin-like growth factor 1 (IGF-1) that activate several cancer intrinsic pathways for survival and proliferation [11,12,13]. Furthermore, obesity induced metabolic stress and lipotoxicity facilitate tumor growth and metastasis through the upregulated activation of oncogenic pathways such as JAK-STAT and NF-κB [13,14]. Obesity-induced tissue hypoxia promotes tumor angiogenesis and metabolic reprogramming by upregulating the expression of hypoxia-inducible factor 1-alpha (HIF-1α) and vascular endothelial growth factor (VEGF) [9,15]. Additionally, adipose-derived mesenchymal stromal cells within the TME undergo phenotypic transition into cancer-associated fibroblasts (CAFs) that secrete growth factors and chemokines to support tumor cell invasion and therapeutic resistance [16,17]. Obesity also significantly impacts immune profiling and function in the TME. For example, obesity-associated inflammation promotes the polarization of tumor-associated macrophages (TAMs) toward an immunosuppressive M2 phenotype, enhances the recruitment and expansion of myeloid-derived suppressor cells (MDSCs) and regulatory T cells (Tregs), and suppresses the cytotoxic activity of natural killer (NK) cells for immunosuppression. Obesity also accelerates the exhaustion of tumor-infiltrating cluster of differentiation 8 (CD8) T cells [18,19,20]. Together, these metabolic, stromal, and immune alterations create a tumor microenvironment that favors tumor initiation, progression, and immune evasion.

Over the past decade, immune checkpoint inhibitors (ICI) have revolutionized cancer treatment by blocking inhibitory immune checkpoint receptors expressed on T cells or their corresponding ligands on tumor cells and antigen-presenting cells to restore anti-tumor immunity [21,22]. Currently, FDA-approved ICIs include antibodies targeting cytotoxic T-lymphocyte associated protein 4 (CTLA-4; ipilimumab, tremelimumab), programmed cell death protein 1 (PD-1; nivolumab, pembrolizumab, and cemiplimab), programmed death-ligand 1 (PD-L1; atezolizumab, durvalumab, and avelumab), and lymphocyte activation gene 3 (LAG-3; relatlimab). These inhibitors, usually used in combination with conventional therapies, have shown significant clinical efficacy in multiple cancer types, such as melanoma, non-small cell lung cancer (NSCLC), renal cell carcinoma (RCC), and hepatocellular carcinoma (HCC) [23,24]. Interestingly, growing clinical evidence has revealed a paradoxical relationship between obesity and ICI efficacy—a relationship referred to as the obesity paradox. This concept refers to the observation that although obesity generally promotes cancer development and progression, clinical studies have reported that obese patients experience improved responses or survival following immune checkpoint blockade compared to patients with normal BMI [25,26]. This paradox has been reported in cancers such as melanoma, non-small cell lung cancer, and renal cell carcinoma, although findings remain inconsistent across tumor types and patient cohorts [27,28]. These observations suggest that obesity may simultaneously promote tumor aggressiveness and modulate anti-tumor immunity in ways that alter responsiveness to immunotherapy. However, the clinical conditions under which this benefit occurs, as well as the biological mechanisms linking obesity to altered ICI response, remain incompletely understood. Clarifying these mechanisms is particularly important given the heterogeneous effects of obesity on tumor immunity and the increasing use of ICIs across cancer types.

In this review, we highlight the obesity paradox as an important and emerging topic in cancer research. We will summarize the current key clinical evidence and experimental studies on the effects of obesity on immune checkpoint inhibitor outcomes, with a specific emphasis on meta-analyses when available. We further discuss potential mechanisms through which obesity may positively or negatively influence ICI efficacy across different malignancies. Finally, we outline key knowledge gaps and propose future research directions to better understand the interplay between obesity and cancer immunotherapy.

## 2. Results

This narrative review includes literature searches conducted in PubMed for studies published through the end of 2025. Searches used combinations of terms related to obesity and body composition, including “obesity,” “BMI,” and “body mass index” together with terms related to cancer immunotherapy and treatment response, including “immunotherapy,” “immune checkpoint inhibitor,” “immune checkpoint blockade,” “treatment response,” and “obesity paradox.” For the clinical evidence summarized in Section 2.1, we included relevant clinical and cohort studies evaluating associations between obesity/BMI/body composition and outcomes following any type of cancer immunotherapy. For Section 2.2, primary research articles and systemic review/meta-analysis were selected to summarize mechanistic findings that may explain the observed clinical associations between obesity and immunotherapy response.

### 2.1. Obesity and Response to ICI Therapy

Since the advent of ICI therapy for cancer treatment, many studies have been conducted to identify markers to predict which patients are likely to respond and those who may not, as only a subset of patients respond. These markers include cancer cell-intrinsic markers such as PD-L1, tumor mutational burden (TMB), microsatellite instability (MSI), and TME markers such as immune cell infiltration [29,30,31]. An early observation was that patients with obesity appeared to obtain greater benefit from ICI therapy than patients without obesity. Subsequent studies have investigated this association further, but the findings have been inconsistent and appear to vary across cancer types. Here, we will summarize studies on the relationship between obesity and response to immunotherapy in different types of cancer (Table 1).

#### 2.1.1. Pan-Cancer and Multi-Cancer

Several large-scale studies comprising multiple cancer types have established the obesity paradox across diverse cancer populations treated with ICIs. Cortellini et al. (2019) provided early evidence from a multicenter study of 976 patients with NSCLC, melanoma, RCC, and other tumor types treated with anti-PD-1/PD-L1 inhibitors. This study found that overweight/obese patients (BMI ≥ 25) had significantly better objective response rates, progression-free survival (PFS) (hazard ratio (HR): 0.46, *p* < 0.0001), and overall survival (OS) (HR: 0.33, *p* < 0.0001), compared with non-overweight patients [32]. In 2022, Yoo et al. published a study evaluating pretreatment BMI in 1840 patients across 16 cancer types treated with ICIs at Memorial Sloan Kettering Cancer Center. They revealed that patients with obesity had significantly longer PFS (HR: 0.86; 95% confidence interval (CI): 0.76–0.97) and OS (HR: 0.81; 95% CI: 0.71–0.93), compared with normal-weight patients in a dose-dependent manner across BMI categories [33]. Xu et al. (2024) conducted a retrospective study of 224 patients from Guangdong Provincial People’s Hospital, integrating BMI with CT-derived body composition metrics including skeletal muscle index (SMI), subcutaneous fat index (SFI), and visceral fat index (VFI), for a multidimensional analysis. Their results further indicate an association between obesity and better overall survival in response to ICI therapy and that this phenomenon is particularly pronounced in male patients and in those with visceral fat accumulation. In addition to body composition, they added that systemic inflammation, reflected by the elevated neutrophil-to-lymphocyte ratio (NLR), was independently associated with poorer survival [28].

Most recently, Alden et al. (2025) prospectively collected serial blood samples from a pan-cancer ICI cohort and found that obese patients had significantly better PFS (HR: 0.44; *p* = 0.01) and OS (HR: 0.24; *p* = 0.02). They additionally revealed that obese patients had lower serum levels of inhibitory cytokines (IL-6, IL-8, IL-15) and higher T-cell exhaustion marker expression at baseline, suggesting that ICI may more effectively reverse obesity-driven T-cell dysfunction [34]. Furthermore, Mastrolonardo et al. (2025) conducted the largest real-world cohort study to date using propensity score-matched electronic health record (EHR) data from the TriNetX database to compare ICI outcomes between obese and normal-BMI patients across multiple solid tumor types. According to their analysis, obesity was associated with significantly improved OS at all time points up to 5 years (HR: 0.69; 95% CI: 0.66–0.72), with subgroup analyses confirming the benefit across most tumor sites, with the notable exception of RCC [27].

#### 2.1.2. Melanoma

Melanoma was the first cancer for which immune checkpoint inhibitors were approved and remains one of the cancers with the best response rate to ICI therapy. Several studies have indicated that obesity may be correlated with better response to ICI therapy in melanoma. The landmark retrospective multicohort analysis by McQuade et al. (2018) examined 1918 patients across six independent cohorts treated with targeted therapy, immunotherapy, or chemotherapy. In the pooled analysis, obesity was associated with improved PFS (adjusted HR: 0.75; 95% CI: 0.56–1.00) and OS (adjusted HR: 0.64; 95% CI: 0.47–0.86) compared with normal BMI. Additionally, this survival benefit was reported to be restricted to patients receiving targeted therapy and immunotherapy and was solely significant in obese males (HR for OS: 0.53; 95% CI: 0.40–0.70) [35]. Richtig et al. (2018) conducted a multicenter observational study of 76 metastatic melanoma patients treated with ipilimumab monotherapy across three centers in Austria, finding that overweight patients (BMI ≥ 25) had significantly higher response rates and a trend toward longer OS; however, no significant PFS difference was observed [36]. Another retrospective cohort study by Naik et al. (2019) exploring the association between BMI, sex, and serum creatinine in melanoma patients treated with immune checkpoint inhibitors found a correlation between BMI and mortality and OS. However, this relationship was restricted to overweight (BMI 25–29.9) and Class I obesity (BMI 30–34.9); they found no correlation with normal weight (BMI < 25) or Class II/III obese (BMI ≥ 35) patients. Furthermore, this association was additionally modulated by gender (only in males) and renal function markers, such as higher serum creatinine levels indicating higher skeletal muscle mass [37]. Jan et al. (2024) examined BMI and T2DM as modifiers of ICI outcomes in a retrospective multicenter melanoma cohort. According to their study, overweight patients had significantly longer OS than non-overweight patients, while type 2 diabetes mellitus was associated with worse OS [43]. Donnelly et al. (2019) examined the relationship between BMI and PFS in metastatic melanoma patients receiving immunotherapy and found an association between obesity and improved PFS; however, this correlation was not statistically significant. This study also found that obesity was associated with a survival benefit in melanoma patients only in the context of receiving immunotherapy combined with other therapies [40].

In contrast to these studies, several studies have found no correlation between obesity and response to immunotherapy in melanoma. Rutkowski et al. (2020) conducted a large multicenter international retrospective study of metastatic melanoma patients receiving targeted therapy (mitogen-activated protein kinase inhibitors (MAPKi)) or immunotherapy. In this study, they reported that elevated BMI has no effect on patients’ PFS and OS [38]. Young et al. (2020) conducted a cohort study with 287 melanoma patients treated with ICIs, focusing more on CT-based body composition than BMI itself. They found that BMI was not associated with clinical benefit or toxicity, while some body composition features were modestly associated with worse outcomes, such as high total adipose tissue index in women [39]. In 2022, Yeung et al. published a retrospective single-center study of 409 patients with advanced unresectable/metastatic cancer (including a melanoma subgroup) treated with ICIs in Canada. The study suggests that obesity was not linked with immunotoxicity, and BMI was not associated with treatment response or overall survival in melanoma patients receiving ICI therapy [42]. Lee et al. (2022) investigated the mechanistic underpinnings of the obesity paradox in melanoma by analyzing visceral adiposity and systemic inflammation in a retrospective cohort of patients with unresectable or metastatic melanoma receiving ICIs. Their findings suggested that obesity appeared protective in standard models, but that the BMI effect largely disappeared after accounting for VFI or systemic immune-inflammation index (SII). Higher VFI was associated with better survival, whereas higher SII predicted worse survival [41].

#### 2.1.3. Non-Small Cell Lung Cancer (NSCLC)

Immunotherapy, alone or in combination with other therapies such as chemotherapy, is currently the standard of care for advanced NSCLC and has been associated with improved 5-year survival rates. Data also support the use of immunotherapy for early-stage NSCLC [69]. Studies have indicated a complex relationship between obesity and response to immunotherapy in NSCLC, with several studies finding a correlation depending on how body composition was measured (BMI, SFI, etc.), whereas others found no correlation. Kichenadasse et al. (2020) performed a pooled post hoc analysis of 2110 patients with advanced NSCLC enrolled in four randomized trials comparing atezolizumab with docetaxel. The authors found that obese patients had significantly improved OS with atezolizumab compared with normal-BMI patients (HR: 0.64; 95% CI: 0.51–0.81), and overweight patients also showed benefit (HR: 0.81; 95% CI: 0.68–0.95). The strength of this association was further enhanced by PD-L1 positivity: obese patients with high PD-L1 expression (≥50% of tumor cells or ≥10% of immune cells) had an OS HR of 0.36 (95% CI: 0.21–0.62). There was no equivalent survival benefit by BMI in the chemotherapy arm, supporting an ICI-specific treatment effect [44]. In 2020, Cortellini et al. reported a study of a large multicenter real-world cohort of NSCLC patients receiving first-line pembrolizumab with PD-L1 expression ≥50%, with an external validation dataset. They observed that overweight and obese patients had significantly higher objective response rates (ORR), as well as longer PFS and OS, compared to normal-weight patients, and this association was not observed in the matched platinum-based chemotherapy arm. They further demonstrated that BMI loss during treatment was associated with poorer outcomes [46]. Recently, Lee et al. (2023) supported a correlation between obesity and response to immunotherapy based on a large retrospective cohort of 820 advanced NSCLC patients. They discovered that obese patients exhibited significantly longer OS compared to non-obese patients, even after adjusting for SMI and VFI [48]. In a multicenter retrospective study of 513 NSCLC patients treated with PD-1/PD-L1 inhibitors, Ichihara et al. (2020) reported a more context-dependent relationship between BMI and immunotherapy outcomes. Although BMI was not associated with PFS or OS in patients receiving first-line pembrolizumab for PD-L1–high tumors, higher BMI was significantly associated with improved PFS and OS in patients receiving ICIs in the second- or later-line setting [45]. These findings suggest that the prognostic value of BMI may vary depending on treatment line and disease context.

In contrast to these studies, Tateishi et al. (2022) conducted a retrospective analysis of 324 NSCLC patients treated with anti–PD-1 inhibitors and found no significant differences in ORR, PFS, or OS across BMI categories [47]. Nie et al. (2024) also published a study of a multicenter cohort of 7021 advanced NSCLC patients treated with chemotherapy, immunotherapy, or chemoimmunotherapy in which they found no association between BMI and OS and PFS in patients receiving immunotherapy-based regimens [49].

Beyond BMI, several studies focused on measures of body composition to further explore the relationship between obesity and immunotherapy outcomes. Degens et al. (2021) analyzed 106 patients with advanced NSCLC treated with second-line nivolumab and found that early weight loss (>2% within six weeks) was independently associated with worse OS. Notably, the weight loss primarily reflected loss in adipose tissue rather than skeletal muscle, suggesting that dynamic changes in body composition may be more clinically relevant than baseline BMI [70]. Makrakis et al. (2023) conducted a prospective study of 52 metastatic non-oncogene-driven NSCLC patients treated with PD-1/PD-L1 targeted therapies and assessed CT-based parameters. The study results showed that SFI was significantly higher among ICI responders, while low lumbar SMI (LSMI) and low SFI predicted worse survival [50]. In a broader imaging-based analysis including NSCLC patients, Decazes et al. (2023) demonstrated that low BMI, low fat body mass (FBM), low subcutaneous fat mass (SFM), and weight loss were associated with poor survival in patients receiving ICIs. They concluded that combining these parameters improved prognostic stratification, again highlighting the interaction between adiposity and muscle depletion in shaping treatment outcomes [71].

#### 2.1.4. Renal Cell Carcinoma (RCC)

RCC consists of 3 main subtypes, papillary, chromophobe, and clear cell, with clear cell RCC (ccRCC) being the most common (70–75%) and the most aggressive [72]. The first-line therapy for metastatic ccRCC is immunotherapy in combination with tyrosine kinase inhibitors that target angiogenesis. As obesity is a known risk factor for both the incidence and progression of RCC [73,74], many studies have investigated the relationship between obesity and response to immunotherapy. Sanchez et al. (2020) performed body weight measurement and transcriptomic profiling across five independent ccRCC cohorts treated with immunotherapy. Comparing obese (BMI ≥ 30) versus normal-weight patients, the study revealed that obesity was independently associated with improved OS. They also observed that the microenvironment varied by BMI within the tumor and that peritumoral adipose tissue might contribute to the survival advantage in obese patients based on the transcriptomic results [51]. Lalani et al. (2021) conducted a multinational retrospective analysis of 735 patients with metastatic RCC from the International Metastatic RCC Database Consortium (IMDC) treated with PD-1/PD-L1-based ICIs. After adjusting for IMDC risk classifications, demographics, histology, and treatment line, elevated BMI was independently associated with improved OS. Furthermore, among patients with next-generation sequencing data, no differences in genomic alteration frequencies or tumor mutational burden (6.8 vs. 6.8 mutations/Mb, *p* = 0.90) were found between high and low BMI groups, suggesting that the survival benefit of obesity in RCC might be driven by systemic or microenvironmental metabolic factors [52]. Herrmann et al. (2022) did another cohort study by collecting weight on the first day of ICI and at 6 weeks of treatment, along with CT-derived SMI as a measure of sarcopenia, from metastatic RCC patients treated with nivolumab. They confirmed that higher baseline BMI was significantly associated with treatment response at first scan (*p* = 0.036), and patients who gained weight during nivolumab treatment had significantly longer OS compared with those who lost weight (*p* = 0.00028). This study provides crucial evidence for the importance of baseline BMI and dynamic weight change during ICI treatment [53]. Sato et al. (2025) evaluated 183 patients with metastatic RCC receiving first-line therapies, including 63 patients (34%) on ICI-based combination therapy and the remainder on tyrosine kinase inhibitor monotherapy, at the Cancer Institute Hospital in Tokyo. Using Japanese population-appropriate BMI cutoffs (≥22 kg/m^2^ for men and ≥18 kg/m^2^ for women to define overweight), they noted that overweight status was independently associated with favorable OS in mRCC patients receiving first-line therapies and ICIs [54]. In a similar vein, De Giorgi et al. (2019) conducted a prospective study with a cohort of patients with metastatic RCC treated with nivolumab between July 2015 and April 2016. They stratified patients as high BMI (≥25) or normal BMI (<25) and included markers for inflammation. They found that patients with a normal BMI and inflammation had 3× the risk of death, indicating that these biomarkers may be prognostic factors for RCC patients treated with nivolumab [55]. In contrast to studies suggesting a beneficial obesity paradox, Boi et al. reported worse outcomes among patients with advanced RCC and obesity receiving anti-PD-1-based therapy. In a cohort of 73 patients, obesity was associated with significantly shorter PFS (*p* = 0.0448) and OS (*p* = 0.0288). Additionally, analysis of treatment-naïve RCC tumors showed a lower frequency of intratumoral PD-1^high^ CD8^+^ T cells in patients with obesity [56].

#### 2.1.5. Urothelial Carcinoma

Urothelial carcinoma (UC) is a heterogeneous group of cancers arising from the bladder and upper urinary tract. ICI therapy has been approved for the treatment of UC in several clinical settings, including as a first-line therapy for cisplatin-ineligible patients, adjuvant therapy following surgery, and as a preoperative therapy. However, only a limited number of patients show a durable response [75]. A few studies have examined the association between obesity and response to ICI therapy in UC. Huang et al. (2024) examined outcomes in a retrospective cohort (*N* = 215) with metastatic UC treated with at least one cycle of ICI monotherapy. Using an Asia-Pacific BMI cutoff of 25 kg/m^2^, they found that patients with higher BMI had significantly longer median OS than those with BMI <25 kg/m^2^ (21.9 vs. 8.3 months; *p* = 0.021), yet PFS was not significantly different [57]. Martini et al. (2021) and Kim et al. (2025) conducted analyses using CT-based body composition in UC patients treated with ICIs, and both concluded that low SMI and VFI, or sarcopenia and myosteatosis, are associated with inferior clinical outcomes [76,77]. Conversely, Ishihara et al. (2023) reported no significant association between BMI and ICI outcomes in an Asian subcohort of UC patients treated with pembrolizumab [58].

#### 2.1.6. Head and Neck Cancers

Head and neck cancers are a group of cancers that originate from cells in the oral cavity, pharynx, larynx, sinonasal cavity, or the salivary glands. ICI therapy has been approved for the treatment of metastatic or recurrent squamous cell carcinomas of the head and neck (HNSCC), though several clinical studies are ongoing that may expand the scope of their use in head and neck cancers [78]. Only a small number of studies have investigated the correlation between body composition or obesity and response to immunotherapy in head and neck cancers. Mastrolonardo et al. (2024) performed a retrospective population-based cohort study using the TriNetX Global Collaborative Network. This study demonstrated that obesity was associated with significantly improved OS from 6 months up to 5 years after ICI treatment. Obesity was also reported to be associated with lower long-term risks of gastrostomy and tracheostomy dependence [59]. Tan et al. (2024) performed a recent analysis in oral tongue squamous cell carcinoma patients receiving neoadjuvant anti-PD-1 therapy, reporting that obesity was prognostically favorable by contributing to better survival and increased infiltration of PD-1+ T cells [61]. In conjunction with these findings, a study by Zhang et al. (2023) found that being underweight was independently associated with worse OS and PFS in 49 recurrent/metastatic HNSCC patients treated with at least one cycle of second-line pembrolizumab [60].

#### 2.1.7. Endometrial Cancer

Immunotherapy has significantly changed the treatment of advanced and recurrent endometrial cancer (EC), particularly for patients with defective mismatch repair (dMMR), and has become a first-line therapy alone or in combination with chemotherapy. While patients with dMMR show high and durable response rates to ICIs, not all patients respond, and some patients with proficient MMR respond well to ICIs [79]. Thus, identifying additional predictors for ICI response is important. A few studies have investigated whether there is a correlation between obesity and/or body composition and response to ICI therapy in endometrial cancer. Gómez-Banoy et al. (2024) conducted a retrospective analysis of 524 EC patients treated with ICI. Overweight and obese patients had significantly prolonged PFS and OS compared with normal-BMI patients based on the analysis, and elevated visceral adipose tissue (VAT) was identified as the strongest independent predictor for improved PFS with ICI therapy. They also reported a higher incidence of immune-related adverse events (irAEs) in obese patients, which indicates the link between improved ICI efficacy and greater immune activation or toxicity [62]. Bernard et al. (2024) performed a retrospective study in patients with recurrent or advanced dMMR/MSI-high (MSI-H) endometrial adenocarcinoma treated with pembrolizumab. They found similar results, reporting that obesity was associated with increased rates of disease control and significantly improved PFS [63]. Most recently, Patterson et al. (2026) conducted another study of advanced or recurrent endometrial cancer patients treated with pembrolizumab at the University of North Carolina. However, this study reported that obesity was associated with worse PFS, and that patients with proficient MMR tumors and obesity had the lowest response rates among all subgroups [64].

#### 2.1.8. Other Cancers

Several other types of cancer have a limited number of studies looking at a correlation between obesity/body composition and response to ICIs. Chen et al. (2022) retrospectively analyzed 101 patients with hepatocellular carcinoma treated with anti–PD-1 therapy. The study found that overweight patients had a higher disease control rate and significantly longer mean PFS than non-overweight patients (10.23 vs. 6.85 months), and BMI remained an independent predictor of PFS on multivariable analysis (HR 0.47, *p* = 0.044) [65]. In contrast, Vithayathil et al. (2023), studying 191 consecutive patients from seven centers who received atezolizumab plus bevacizumab for HCC, reported that BMI was not associated with ICI efficacy: median OS (15.1 vs. 14.9 months), PFS (7.1 vs. 6.1 months), ORR, and Disease control rate (DCR) were all similar between BMI groups in their cohort [66]. Among colorectal cancer (CRC) patients, ICI therapy is primarily effective in MSI-H or dMMR tumors. So far, only one retrospective study by Zeng et al. (2025) examined dMMR/MSI-H CRC patients receiving neoadjuvant PD-1 blockade and found that lower BMI and lower VAT were linked to poorer pathological response, while higher VAT was associated with higher pathological complete response rates [67]. For breast cancer, ICI therapy has been approved for triple-negative breast cancer, though response rates are relatively low, and predictors of response are critically needed [80,81]. Chiang et al. (2025) performed a retrospective cohort study using the TriNetX Analytics Network on 1628 adult female breast cancer patients who received pembrolizumab. When matched with other demographic and medical conditions, the study indicates that patients with BMI ≥25 kg/m^2^ had a significantly lower risk of 1-year all-cause mortality than those with BMI <25 kg/m^2^ (HR: 0.51; 95% CI: 0.33–0.81). However, no detailed tumor subtype-specific efficacy endpoints such as ORR or PFS were reported in this paper [68]. Interestingly, mouse models of obesity-driven breast cancer have shown that diet-induced obesity increases response to ICIs [82,83]. More comprehensive studies need to be performed to draw a clearer picture of how obesity affects ICI response in these cancers.

### 2.2. Obesity Associated Immune Dysfunction in Cancer and Obesity Paradox: Biological Mechanisms

Growing clinical and translational evidence suggests a strong association between obesity and immune dysfunction in the context of cancer [18,84,85,86]. To model conditions of obesity and metabolic syndrome, most translational studies use diet-induced obesity (DIO) rodent models, which typically involve feeding animals a high-fat diet (HFD) containing 40–60% fat for 5–30 weeks [87]. Ringer et al. reported increased tumor growth in HFD-fed mice compared with mice fed a corresponding control diet (CD) across multiple immunogenic tumor models, including MC38 colorectal adenocarcinoma, E0771 breast adenocarcinoma, and B16 melanoma. Subsequent analyses revealed a lower proportion of tumor-infiltrating CD8+ T cells, along with evidence of reduced proliferative and effector capacity, in HFD tumor-bearing mice compared with the CD group [20]. Consistent with these findings, Bader et al. reported similar results and additionally identified an increased proportion of PD-1+ tumor-associated macrophages (TAMs) in HFD tumor-bearing mice. These macrophages exhibited impaired phagocytic activity and antigen-presenting function [82]. Another study found that B16 melanoma and 4T1 breast carcinoma tumors grew more rapidly in DIO mice and were associated with an increased proportion of tumor-infiltrating PD-1+ CD8+ T cells. Furthermore, T cells from tumor-bearing DIO mice exhibited transcriptional profiles consistent with exhaustion and reduced effector function, including marked upregulation of genes associated with cellular senescence and inflammation-induced anergy [88]. Collectively, these studies strongly suggest a direct relationship between obesity and alterations within the tumor microenvironment (TME) that promote immune dysfunction and enhance tumor growth.

Mechanistically, it has been proposed that leptin, a peptide hormone produced primarily by adipose tissue and a key pro-inflammatory mediator associated with obesity, may directly contribute to immune dysfunction and T-cell exhaustion [88,89]. In vitro, treatment with recombinant mouse leptin increased PD-1 expression on CD8 T cells through a STAT3-dependent signaling pathway. Similarly, leptin-deficient mice bearing B16 tumors that were treated with recombinant leptin exhibited increased tumor growth and impaired T-cell function, characterized by a higher proportion of PD-1+ CD8 T cells, reduced interferon-γ (IFN-γ) production, and decreased expression of proliferation markers [88]. In addition, leptin and other obesity-associated adipokines and cytokines, including IFN-γ, IL-6, IL-1β, TNF, and insulin, have been shown to induce PD-1 expression in bone marrow-derived macrophages. Furthermore, in vitro treatment with saturated fatty acids, which mimics the elevated levels of circulating free fatty acids observed in obesity, resulted in increased macrophage toxicity and PD-1 expression [82]. Finally, preclinical studies have demonstrated that, under obese conditions, tumor cells can undergo intrinsic metabolic reprogramming characterized by increased free fatty acid mobilization and oxidation, thereby depleting the TME of fatty acids that may be essential for effective antitumor immunity, particularly CD8 T-cell-mediated tumor control [20].

Multiple studies have also reported observations consistent with the “obesity paradox,” in which DIO tumor-bearing mice respond more effectively to ICIs than their control diet counterparts. Mechanistically, ICI therapies such as anti-PD-1 can restore CD8+T-cell activity in DIO mice, leading to increased CD8 T-cell infiltration, a higher CD8:CD4 ratio, and enhanced effector function, as reflected by increased production of TNF-α and IFN-γ [88]. In addition, the increased prevalence of PD-1+ TAMs may contribute to the obesity paradox, as ICI therapy could reinvigorate key phenotypic functions of this myeloid subset, including antigen presentation, thereby enhancing T-cell-mediated immune surveillance [82]. Finally, melanoma has been shown to acquire resistance to ICI through the development of a “hypermetabolic” state characterized by increased glycolysis and oxidative phosphorylation, which creates a nutrient-depleted TME that renders CD8 T cells dysfunctional [90]. In this context, Hahn et al. demonstrated that overweight and obese melanoma patients exhibited signatures of reduced oxidative phosphorylation (OXPHOS) and glycolysis. These findings suggest that the improved responses to ICI therapies observed in obese melanoma patients may be related to a more metabolically quiescent tumor state and a more immune-permissive TME [91]. In another study, Dyck L. et al. found that obesity results in a decrease in glutamine uptake in tumor-infiltrating CD8 T cells, leading to decreased mTOR signaling. This results in decreased CD8 T cell proliferation and expression of chemokines and the chemokine receptor C-X-C motif chemokine receptor 3 (CXCR3), which play a vital role in the trafficking of CD8 T cells to tumors. Moreover, treatment of obese mice with anti-PD-1 rescued the metabolic dysfunction in T cells, suggesting that ICIs may also function by metabolic reinvigoration of CD8 T cells [92]. Other studies have found that obesity skews T cells towards lipid metabolism. Excess lipid metabolism can lead to lipotoxicity, resulting in endoplasmic reticulum stress and mitochondrial dysfunction, leading to T cell exhaustion and apoptosis [93,94,95]. Whether the change in lipid metabolism in T cells during obesity is affected by ICI therapy or whether this process is involved in the increased response rate to ICI therapy observed in obese patients needs further study. Several studies have shown that targeting lipid metabolism in CD8 T cells can increase antitumor immunity and improve immunotherapy efficacy [96,97,98,99].

## 3. Discussion

The clinical studies reviewed here suggest that obesity and body composition may influence outcomes following ICI therapy, although this association is heterogeneous and should not be interpreted as a uniform benefit of obesity. Several large pan-cancer cohorts have reported improved survival among patients with elevated BMI receiving ICIs, supporting the clinical observation of the “obesity paradox” [27,32,33]. However, the magnitude of this association varies considerably across studies, and null or opposing findings have also been reported within individual cancer types. Collectively, these observations suggest that the relationship between obesity and treatment outcome appears to be complex, involving the interplay of different factors depending on tumor type, body composition, sex, systemic inflammation, and treatment-related factors.

Studies that include non-ICI comparator groups provide particularly useful evidence for distinguishing whether obesity is associated specifically with response to ICIs or simply with prognosis. In analyses of randomized trials comparing atezolizumab with docetaxel in non-small cell lung cancer (NSCLC), Kichenadasse et al. observed an association between higher BMI and improved survival in patients receiving atezolizumab but not docetaxel, with a stronger association among patients with PD-L1–positive tumors [44]. Similarly, Cortellini et al. reported improved outcomes associated with elevated BMI in patients receiving first-line pembrolizumab, whereas a comparable association was not observed in a matched cohort receiving platinum-based chemotherapy [46]. These findings raise the possibility that metabolic or immune alterations associated with obesity may interact with pathways targeted by ICIs. Nevertheless, these observations remain insufficient to establish obesity as a predictive biomarker, particularly given the retrospective nature of most analyses and the potential for residual confounding.

The consistency of the association also differs among tumor types. Melanoma, NSCLC, and renal cell carcinoma (RCC) have been studied most extensively, in part because of their long clinical history of ICI use. In NSCLC, several studies support an association between elevated BMI and improved ICI outcomes, including the comparator studies described above, although other large cohorts have reported no significant association [44,46,49]. Studies in RCC have more consistently reported favorable outcomes among patients with elevated BMI or greater adiposity [51,52,53,54,55]. Interpretation in RCC is complicated, however, by the previously recognized association between obesity and improved RCC prognosis independent of immunotherapy. Melanoma studies are particularly controversial, with several cohorts reporting improved outcomes associated with obesity and others showing little or no association [34,35,36,37,38,39,42,48]. For other malignancies, including urothelial, head and neck, endometrial, hepatocellular, colorectal, and breast cancers, the available studies remain comparatively limited and heterogeneous. Thus, current evidence does not assume that the obesity paradox operates similarly across malignancies.

An additional challenge is that BMI may not adequately represent the biological features responsible for these associations. BMI does not distinguish adipose tissue from skeletal muscle and provides no information regarding fat distribution, muscle mass, or cancer-associated weight loss. Studies incorporating CT-based measures of body composition therefore provide important context. In melanoma, for example, Lee et al. found that associations between BMI and survival were substantially influenced by visceral adiposity and systemic inflammation, while Young et al. reported relationships with specific measures of adiposity despite finding little association with BMI itself [39,48]. Similarly, studies in NSCLC and urothelial carcinoma have identified sarcopenia, low skeletal muscle mass, or myosteatosis as adverse prognostic features during ICI therapy [50,71,76,77]. These findings suggest that at least part of the apparent BMI-associated survival advantage may reflect differences in body composition, nutritional status, or cancer-associated cachexia rather than adiposity alone. Thus, additional assessment of adipose and skeletal muscle compartments may be more informative than BMI alone when evaluating the relationship between obesity and immunotherapy response.

Other patient- and treatment-specific factors may further contribute to the inconsistent findings across studies. Sex is one potential effect modifier. Several melanoma studies have reported that the association between obesity and improved ICI outcomes is more apparent in male patients, and sex-dependent associations have also been observed in pan-cancer analyses [35,37,68]. Differences in adipose distribution, sex hormones, metabolism, and immune regulation depending on sex may contribute to these observations, although the underlying mechanisms remain unclear. Differences in how obesity is defined also complicate comparisons across cohorts, as studies have used various BMI thresholds [54,57]. Treatment heterogeneity represents another important consideration. Studies have included patients receiving ICIs in different lines of therapy and have variably combined anti-PD-1, anti-PD-L1, anti-CTLA-4, and ICI-based combination regimens. In some cases, the association between BMI and outcome has differed according to treatment line; for example, Ichihara et al. observed a BMI-associated benefit in patients receiving later-line therapy but not among patients receiving first-line treatment for PD-L1–high disease [45]. Such differences may contribute to apparently conflicting results and emphasize the importance of accounting for treatment setting when evaluating obesity as a potential modifier of ICI response. As such, what is evident from a review of these studies is that the relationship between obesity and immunotherapy response is context-dependent, depending on several factors including cancer type, combinatorial therapy, what indices are used to measure obesity, sex, and inflammatory markers. More studies, particularly meta-analyses, need to be performed to get a better understanding of under what contexts obesity can be used as a predictor for immunotherapy response.

Mechanistically, obesity could influence immunotherapy response through several interconnected changes within the TME. Obesity is associated with chronic inflammation, altered adipokine signaling, metabolic dysregulation, and impaired antitumor immunity. Preclinical studies have demonstrated reduced CD8 T-cell infiltration and function, increased expression of PD-1, altered T-cell metabolism, and changes in TAM phenotypes in obese hosts (Figure 1) [20,82,88,92]. However, studies showing a mechanistic link between obesity and response to ICI are limited. The simplest explanation for the obesity paradox is that obesity-associated immune dysfunction and immunosuppression lead to an increase in immune checkpoint inhibitor pathways, creating a state in which inhibiting these pathways increases anti-tumor immunity. For example, Wang et al. demonstrated that obesity promoted PD-1 expression and T-cell dysfunction through leptin-associated signaling in preclinical models and that obese mice showed enhanced responsiveness to anti-PD-1 therapy [88]. However, evidence for its role as a prognostic indicator of response to ICI therapy in humans is lacking, and several studies have found that leptin can promote an immunosuppressive effect in tumors or enhance anti-tumor immune responses depending on the context [100,101,102]. Emerging evidence suggests that the mechanisms linking obesity to ICI response may differ according to tumor context, although the relative contribution of individual pathways remains poorly defined. In NSCLC, relatively direct mechanistic evidence supports a metabolic–immune axis in which obesity-associated increases in tumor glycolysis and lactate promote MCT1-dependent histone lactylation and PD-1 expression in CD8 T cells, potentially increasing susceptibility to PD-1 blockade [103]. In contrast, studies in RCC have emphasized obesity-associated myeloid inflammation, where increased IL-1β signaling and MDSC accumulation can suppress antitumor immunity and reduce responsiveness to anti-PD-1 therapy [56]. In melanoma, both increased checkpoint-associated T-cell dysfunction and alterations in tumor metabolism have been proposed [25]. Thus, rather than supporting a single mechanism underlying the obesity paradox across cancers, current evidence suggests that metabolic, lymphoid, and myeloid effects of obesity may contribute differently depending on the TME; however, whether these differences reflect true tumor-specific biology or differences in the mechanisms investigated to date remains unclear. It is possible that some or all of these mechanisms are involved in how obesity affects the response to immunotherapy depending on the context (Figure 1).

Several limitations of the existing literature should be considered when interpreting these findings. Most studies examining obesity and ICI outcomes are retrospective, and many are single-center analyses with relatively small patient populations. Adjustment for major prognostic variables is inconsistent, and differences in cancer stage, molecular subtype, systemic inflammation, treatment line, and concomitant therapies may contribute to residual confounding [34,44,50,55]. Furthermore, both obesity and treatment outcome have been defined differently across studies, limiting direct comparisons. Future studies would benefit from standardized assessment of body composition using imaging-based measures in addition to BMI, longitudinal evaluation of weight and muscle loss, and incorporation of systemic metabolic and inflammatory markers. Stratification by sex, cancer subtype, checkpoint target, treatment line, and combination regimen will also be important for determining the clinical contexts in which obesity or specific components of body composition are associated with ICI efficacy.

As evident from the studies reviewed here, the relationship between obesity/body composition and response to immunotherapy in cancer is highly context-dependent and varies according to cancer type, sex, body-composition measures, inflammatory status, and treatment setting. There may be numerous mechanisms involved in this relationship, independently or working together depending on the context. Thus, obesity may not be a simple prognostic marker for response to immunotherapy but rather a complex condition that modifies the tumor immune microenvironment. Future studies should look for ways to use obesity-associated metabolic and immune dysfunction to identify therapeutic targets and modalities to improve therapeutic outcomes. To accomplish this, a better understanding of the relationship between obesity and ICI response under different contexts and the mechanisms for this relationship are required. Defining these mechanisms could ultimately identify metabolic or immune vulnerabilities that can be therapeutically targeted to improve the efficacy of immunotherapy across broader patient populations.

## Figures and Tables

**Figure 1:**
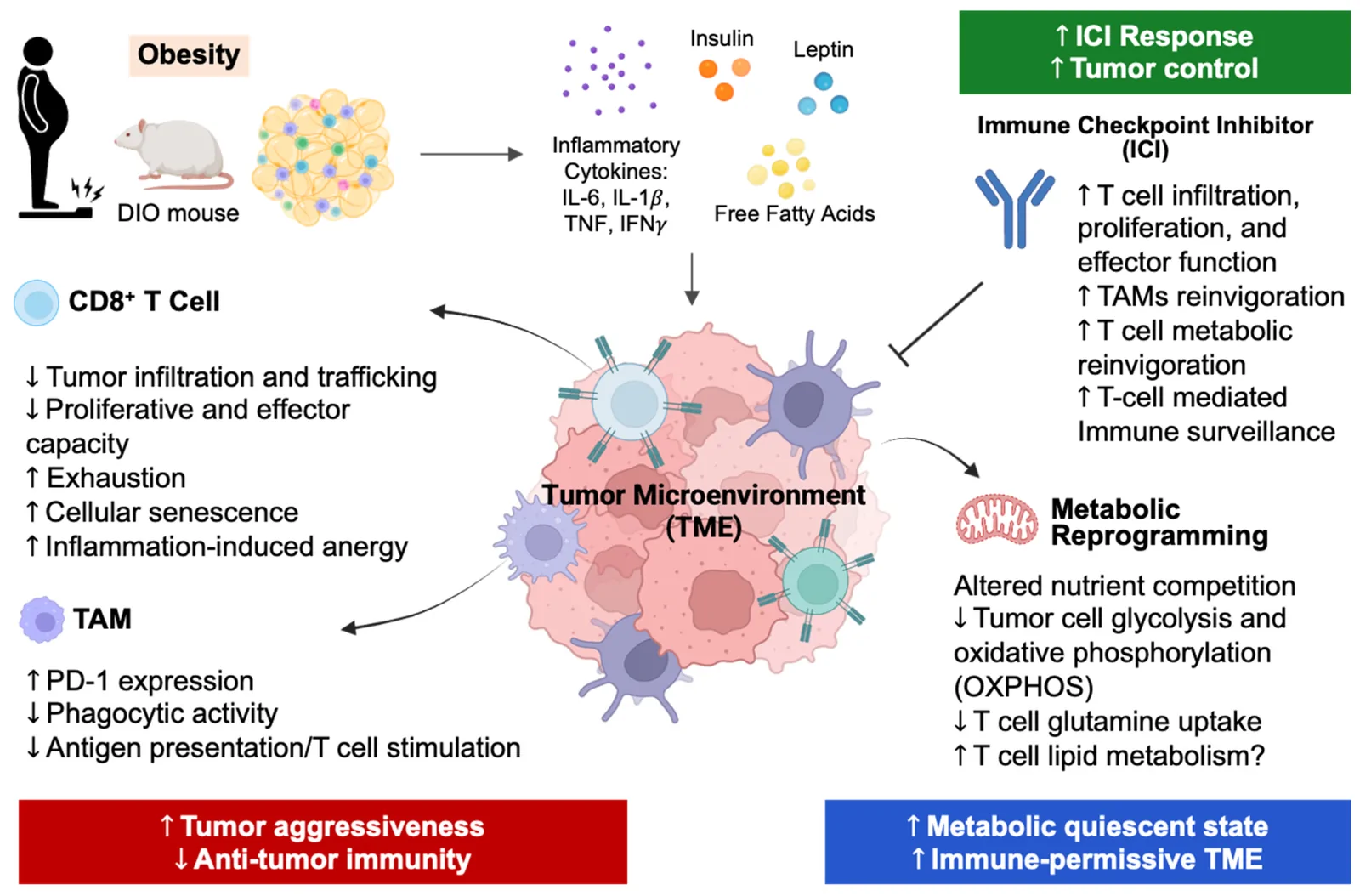
**Proposed mechanisms linking obesity to altered antitumor immunity and immune checkpoint inhibitor (ICI) response.** Obesity promotes systemic metabolic and inflammatory changes that reshape the tumor microenvironment, impair CD8^+^ T-cell function, alter tumor-associated macrophage activity, and induce metabolic reprogramming. These changes may contribute to tumor progression while also generating an immune state that can be partially reversed by ICI therapy, resulting in enhanced T-cell activity, immune surveillance, and tumor control. DIO: diet-induced obesity; IL: interleukin; TNF: tumor necrosis factor; IFN: interferon; TAMs: tumor-associated macrophages. Created in biorender. Wang, L. (2026) https://BioRender.com/nrl38sx (accessed on 3 September 2026).

**Table 1:** Clinical evidence on the association between obesity and outcomes following immune checkpoint inhibitor treatment, organized by cancer type. Studies are listed by author and publication year, study design and sample size, BMI category, and reported quantitative outcomes, including overall survival, progression-free survival, mortality, and treatment response. Studies discussed in the text that did not evaluate BMI as the exposure were excluded from the table.

**Pan-Cancer and Multi-Cancer**			
**Publication**	**Study Type (Sample Size)**	**BMI Category**	**Results**
Cortellini et al. (2019) [32]	Retrospective (N = 976)	Overweight/obese (BMI ≥ 25); Non-overweight (BMI < 25).	Obese patients have better survival: TTF HR: 0.51 (95% CI: 0.44–0.60), *p* < 0.0001; PFS HR: 0.46 (95% CI: 0.39–0.54), *p* < 0.0001; OS HR: 0.33 (95% CI: 0.28–0.41), *p* < 0.0001.
Yoo et al. (2022) [33]	Retrospective (N = 1840)	Obese (BMI ≥ 30); Overweight (BMI 25–29.9); Normal weight (BMI 18.5–24.9).	Obese patients have better survival: OS HR: 0.81 (95% CI: 0.71–0.93); PFS HR: 0.86 (95% CI: 0.76–0.97).
Xu et al. (2024) [28]	Retrospective (N = 224)	Obese weight (BMI ≥ 25); Overweight (BMI 23.0–24.9); Normal weight (BMI 18.5–22.9); Underweight (BMI < 18.5); And CT-derived body composition.	Obese patients had a longer OS (mean OS 43.1 months; 95% CI 32.4 to 53.9 months); underweight patients had the shortest OS (mean OS 24.5 months; 95% CI 18.2 to 30.9 months) compared to other patients (log-rank *p* = 0.027).
Alden et al. (2025) [34]	Prospective (N = 94)	Obese (BMI ≥ 30); Normal weight (BMI ≥ 18.5 and < 30).	Obese patients have better survival: PFS HR: 0.44 (95% CI: 0.24–0.81), *p* = 0.01; OS HR: 0.24 (95% CI: 0.07–0.80), *p* = 0.02.
Mastrolonardo et al. (2025) [27]	Retrospective (N = 18,434)	Obese (BMI > 30); Normal weight (BMI 20–24.9).	Obese patients have better survival: OS HR 0.69 (95% CI: 0.66–0.72); respiratory/intrathoracic HR: 0.77 (95% CI: 0.72–0.83); cutaneous HR: 0.62 (95% CI: 0.63–0.78); head and neck HR: 0.67 (95% CI: 0.58–0.78); gastrointestinal HR: 0.67 (95% CI: 0.58–0.78); breast HR: 0.66 (95% CI: 0.55–0.79); genitourinary HR: 0.57 (95% CI: 0.34–0.93).
**Melanoma**			
**Publication**	**Study Type (Sample Size)**	**BMI Category**	**Results**
McQuade et al. (2018) [35]	Retrospective (N = 1918)	Obese (BMI ≥ 30); Overweight (BMI 25–29.9); Normal weight (BMI 18.5–24.9).	Obese patients have better survival: PFS HR: 0.75 (95% CI: 0.56–1.00); OS HR: 0.64 (95% CI: 0.47–0.86); benefit restricted to males.
Richtig et al. (2018) [36]	Retrospective (N = 76)	Overweight (BMI ≥ 25); Normal weight (BMI < 25).	No significant improvement in survival for obese patients: PFS HR: 1.03 (95% CI: 0.62–1.70), *p* = 0.924; OS HR: 1.81 (95% CI: 0.98–3.33), *p* = 0.056.
Naik et al. (2019) [37]	Retrospective (N = 139)	Class II/III obesity (BMI ≥ 35); Overweight/Class I obesity (BMI 25–35); Normal weight (BMI 18.5–25).	Overweight/Class I obesity has better survival: OS HR: 0.26 (95%CI: 0.1–0.71), *p* = 0.001; PFS HR: 0.43 (95%CI: 0.19–0.95), *p* = 0.038; association predominantly in males.
Rutkowski et al. (2020) [38]	Retrospective (N = 417)	Obese (BMI ≥ 30); Overweight (BMI 25–29.9); Normal weight (BMI 18.5–24.9); Underweight (BMI < 18.5).	No impact of BMI on survival: PFS HR: 1.00 (95% CI: 0.98–1.03), *p* = 0.732; OS HR: 1.02 (95% CI: 0.99–1.05), *p* = 0.202.
Young et al. (2020) [39]	Retrospective (N = 287)	Obese (BMI ≥ 30); Overweight (BMI 25–29.9); Normal (BMI < 25); And CT body composition.	No association with BMI: Overweight vs. normal: PFS HR: 1.04 (95% CI: 0.71–1.52), *p* = 0.84; OS HR: 1.10 (95% CI 0.71–1.70), *p* = 0.67; Obese vs. normal: PFS HR 1.28 (95% CI 0.9–1.83), *p* = 0.18; OS HR 1.10 (95% CI 0.72–1.67), *p* = 0.65.
Donnelly et al. (2019) [40]	Prospective (N = 423)	Obese (BMI ≥ 30); Overweight (BMI 25–29.9); Normal (BMI < 25).	No significant improvements in PFS and OS among obese or overweight patients (*p* = 0.75); Survival benefits only in combination ICIs: Obese PFS HR: 0.17 (95% CI: 0.04–0.65), *p* = 0.01; Overweight PFS HR: 0.36 (95% CI: 0.15–0.85), *p* = 0.02.
Lee et al. (2022) [41]	Retrospective (N = 266)	Obese (BMI ≥ 25); Overweight (BMI 23.0–24.9); Normal (BMI 18.5–22.9); Underweight (BMI < 18.5); And CT body composition.	Positive effect of obesity: OS HR: 0.60 (95% CI: 0.37–0.99), *p* = 0.048; but disappeared after adjusting for VFI or SII.
Yeung et al. (2022) [42]	Retrospective (N = 409)	Obese (BMI ≥ 30); Non-obese (BMI < 30).	No association between BMI and OS or treatment response.
Jan et al. (2024) [43]	Retrospective (N = 2078)	Overweight (BMI ≥ 25); Non-overweight (BMI < 25); And T2DM status. Subgroups: Severely obese (BMI ≥ 35); Obese (30 ≤ BMI < 35) Overweight (25 ≤ BMI < 30); Healthy weight (18.5 ≤ BMI < 25); Underweight (BMI < 18.5)	Overweight patients had a better OS: OS HR: 0.69 (95% CI: 0.60–0.80), *p* < 0.001; Overweight (median 67.7 months, obese (median not reached months), or severely obese (median 59.0 months) had higher OS compared with patients who were underweight (median 11.2 months) or healthy weight (median 38.6 months), Log-rank *p* <0.001.
**Non-Small Cell Lung Cancer (NSCLC)**			
**Publication**	**Study Type (Sample Size)**	**BMI Category**	**Results**
Kichenadasse et al. (2020) [44]	Retrospective (N = 2110)	Obese (BMI ≥ 30); Overweight (BMI 25–29.9); Normal weight (BMI 18.5–24.9); Underweight (BMI < 18.5).	Obese patients have better survival: Obese OS HR: 0.64 (95% CI: 0.51–0.81), *p* < 0.001; Overweight OS HR: 0.81 (95% CI: 0.68–0.95), *p* < 0.001; Obese PFS HR: 0.86 (95% CI: 0.73–1.01), *p* = 0.09; Overweight PFS HR: 0.89 (95% CI: 0.78–1.01), *p* = 0.09.
Ichihara et al. (2020) [45]	Retrospective (N = 513)	High (BMI ≥ 22.0); Low (BMI < 22.0).	No association between BMI and patient survival: PFS HR: 0.94 (95% CI: 0.53–1.65), *p* = 0.84; OS HR: 0.67 (95% CI: 0.32–1.40), *p* = 0.29. Higher BMI associated with better survival in patients treated with second- or later-line ICIs.
Cortellini et al. (2020) [46]	Retrospective (N = 962)	Obese (BMI ≥ 30); Overweight (25 ≤ BMI ≤ 29.9); Healthy weight (18.5 ≤ BMI ≤ 24.9); Underweight (BMI < 18.5).	Obese patients have response rate and survival: ORR OR: 1.61 (95% CI: 1.04–2.50), *p* = 0.0208; PFS HR: 0.61 (95% CI: 0.45–0.82), *p* = 0.0012; OS HR: 0.70 (95% CI: 0.49–0.99), *p* = 0.0474.
Tateishi et al. (2022) [47]	Retrospective (N = 324)	Overweight (BMI ≥ 25); Non-overweight (BMI < 25).	No association between BMI and patient response rate and survival: PFS HR: 0.68 (95% CI: 0.29–1.58), *p* = 0.35; OS HR: 0.97 (95% CI: 0.38–2.49), *p* = 0.95.
Lee et al. (2023) [48]	Retrospective (N = 820)	Obese (BMI ≥ 25); Overweight (BMI 23.0–24.9); Normal (BMI 18.5–22.9); Underweight (BMI < 18.5); And body composition imaging.	Obese patients have better survival: OS HR: 0.64 (95% CI: 0.52–0.80); *p* < 0.001; Association remains significant after adjusting for SMI and VFI.
Nie et al. (2024) [49]	Retrospective (N = 7021)	Obese (BMI ≥ 30); Overweight (BMI 25–29.9); Normal weight (BMI 18.5–24.9); Underweight (BMI < 18.5).	No association between BMI and survival in patients: PFS HR: 1.03 (95% CI: 0,87–1.22); HR: 0.92 (95% CI: 0.77–1.09).
Makrakis et al. (2023) [50]	Prospective (N = 52)	Obese (BMI > 30); Overweight (25 ≤ BMI < 30); Normal (BMI < 25); And Intramuscular Fat Index (IMFI), Visceral Fat Index (VFI), Subcutaneous Fat Index (SFI), Lumbar Skeletal Muscle Index (LSMI) categories.	No BMI association reported. Patients with baseline LSMI < LNL experienced worse PFS (3.30 vs. 7.33 months, *p* = 0.040) and OS (6.37 vs. not reached months, *p* = 0.009). Low SFI negatively associated with OS (5.43 vs. 14.03 months, *p* = 0.020).
**Renal Cell Carcinoma (RCC)**			
**Publication**	**Study Type (Sample Size)**	**BMI Category**	**Results**
Sanchez et al. (2020) [51]	Retrospective (N = 256)	Obese (BMI ≥ 30); Normal (BMI 18.5–24.9); And transcriptomics evaluation.	Obese patients have better survival across two cohorts: OS HR: 0.41 (95% CI: 0.22–0.75); OS HR: 0·54 (95% CI: 0.31–0.95); but significance disappeared after adjustment for IMDC score.
Lalani et al. (2021) [52]	Retrospective (N = 735)	High (BMI ≥ 25); Low (BMI < 25); And tumor mutation burden.	Obese patients have better survival: OS HR: 0.75 (95% CI: 0.57–0.97), *p* = 0.03; ORR OR: 1.51 (95% CI: 0.98–2.32), *p* = 0.06.
Herrmann et al. (2022) [53]	Retrospective (N = 46)	Baseline BMI (continuous) + weight change + SMI	Higher baseline BMI associated with response at first scan (*p* = 0.036); Weight gain positively associated with longer OS (*p* = 0.00028).
Sato et al. (2025) [54]	Retrospective (N = 183)	Men: Overweight (BMI ≥ 22); Normal (BMI < 22). Women: Overweight (BMI ≥ 18); Normal (BMI < 18).	Overweight is positively associated with better survival: OS HR: 0.27 (*p* = 0.031).
De Giorgi et al. (2019) [55]	Prospective (N = 313)	High (BMI ≥ 25); Normal (BMI < 25).	Normal BMI associated with worse survival compared to high BMI: OS HR: 1.59 (95% CI: 1.10–2.30). Normal BMI combined with inflammation is critical prognostic factor for OS.
Boi et al. (2020) [56]	Retrospective (N = 73)	Obese (BMI ≥ 30); Non-obese (BMI < 30).	Obesity associated with poorer PFS and OS: PFS HR (Non-obese): 0.54 (95% CI: 0.31–0.95); OS HR (Non-obese): 0.48 (95%CI: 0.24–0.96).
**Urothelial Carcinoma**			
**Publication**	**Study Type (Sample Size)**	**BMI Category**	**Results**
Huang et al. (2024) [57]	Retrospective (N = 215)	Obese (BMI ≥ 25); Non-obese (BMI < 25).	BMI-high patients had significantly better OS than BMI-low patients (21.9 vs. 8.3 months; *p* = 0.021); No significant difference in PFS between the BMI-high and BMI-low groups (4.7 vs. 2.8 months; *p* = 0.16).
Ishihara et al. (2023) [58]	Retrospective (N = 309)	High (BMI ≥ 25); Low (BMI < 25).	No significant association between high BMI and PFS/OS: PFS: median 5.43 months (95% CI: 3.73–14.3 months) vs. median 6.07 months (95% CI: 3.03–9.33 months), *p* = 0.841; OS: median 15.0 months (95% CI: 7.70–21.2 months) vs. median 12.1 months (95% CI: 6.87–21.4), *p* = 0.983.
**Head and Neck Cancers**			
**Publication**	**Study Type** **(Sample Size)**	**BMI Category**	**Results**
Mastrolonardo et al. (2024) [59]	Retrospective (N = 166)	Obese (BMI ≥ 30); Normal (BMI 20.0–24.9).	Obese patients have better survival: 6-month OS HR: 0.54 (95% CI: 0.31–0.96); 3-year OS HR: 0.56 (95% CI: 0.38–0.83); 5-year OS HR: 0.62 (95% CI: 0.44–0.86). Lower long-term risk of gastrostomy and tracheostomy dependence.
Zhang et al. (2023) [60]	Retrospective (N = 49)	Obese (BMI ≥ 30.0); Overweight (25 ≤ BMI < 29.9); Normal weight (18.5 ≤ BMI < 24.9); Underweight (BMI < 18.5).	No significant association between survival and obesity, but underweight patients have poorer survival: OS HR: 6.862 (95% CI:1.566–30.064), *p* = 0.011; PFS HR: 5.672 (95% CI:1.364–23.586), *p* = 0.017.
Tan et al. (2024) [61]	Retrospective (N = 56)	High (BMI ≥ 24); Low (BMI < 24).	High BMI associated with better PFS: PFS HR: 0.015 (95% CI: 0.001–0.477), *p* = 0.015;
**Endometrial Cancer**			
**Publication**	**Study Type** **(Sample Size)**	**BMI Category**	**Results**
Gómez-Banoy et al. (2024) [62]	Retrospective (N = 524)	Obese (BMI ≥ 30); Overweight (BMI 25–29.9); Normal BMI (18.5–24.9).	Obesity associated with improved survival: Obese PFS HR: 0.57 (95% CI: 0.42–0.79), *p* = 0.0005; Obese OS HR: 0.64 (95% CI: 0.45–0.92), *p* = 0.0144; Overweight PFS HR: 0.62 (95% CI: 0.45–0.87), *p* = 0.0052; Overweight OS HR: 0.53 (95% CI: 0.35–0.79), *p* = 0.0020; Elevated VAT was identified as an independent predictor for improved PFS.
Bernard et al. (2024) [63]	Retrospective (N = 44)	Obese (BMI ≥ 30); Non-obese (BMI < 30).	Obesity associated with disease control rate and PFS: Disease control OR: 4.9 (95% CI: 1.09–28), *p* = 0.05; PFS HR: 0.38 (95% CI: 0.148–0.96), *p* = 0.03; OS not significant (*p* = 0.5).
Patterson et al. (2026) [64]	Retrospective (N = 179)	Obese I (BMI > 40); Obese II (BMI 30–40); Normal (BMI < 30).	High BMI associated with worse survival: BMI 30–40 PFS HR: 1.55 (95% CI: 1.02–2.35), *p* = 0.041; BMI > 40 PFS HR: 1.91 (95% CI: 1.13–3.21), *p* = 0.014; No significant differences in OS.
**Hepatocellular Carcinoma (HCC)**			
**Publication**	**Study Type** **(Sample Size)**	**BMI Category**	**Results**
Chen et al. (2022) [65]	Retrospective (N = 101)	Overweight (BMI > 24.9); Non-overweight (BMI ≤ 24.9).	Overweight associated with better PFS and disease control rates: Mean PFS in overweight patients (10.23 months) vs. non-overweight patients (6.85 months; *p* = 0.027); PFS HR: 0.47 (95% CI: 0.22–0.98), *p* = 0.044.
Vithayathil et al. (2023) [66]	Retrospective (N = 191)	Overweight (BMI ≥ 25); Non-overweight (BMI < 25).	No association between BMI and survival: OS HR: 1.00 (95% CI: 0.60–1.64), *p* = 0.98; PFS HR: 0.70 (95% CI: 0.41–1.20), *p* = 0.20.
**Colorectal Cancer (MSI-H/dMMR)**			
**Publication**	**Study Type** **(Sample Size)**	**BMI Category**	**Results**
Zeng et al. (2025) [67]	Retrospective (N = 84)	High (BMI ≥ 24); + body composition categories	No association between BMI and treatment response rate; Low BMI and VAT linked to poorer pathological response. No survival analysis.
**Breast Cancer**			
**Publication**	**Study Type** **(Sample Size)**	**BMI Category**	**Results**
Chiang et al. (2025) [68]	Retrospective (N = 1628)	Obese/overweight (BMI ≥ 25); Normal weight (BMI < 25).	Obese/overweight associated with low mortality risk: 1-year all-cause mortality HR: 0.51 (95% CI: 0.33–0.81).

## Data Availability

No new data were created or analyzed in this study. Data sharing is not applicable to this article.

## Abbreviations

The following abbreviations are used in this manuscript:

ICIs	immune checkpoint inhibitors
BMI	body mass index
WHO	World Health Organization
NAFLD	non-alcoholic fatty liver disease
TME	tumor microenvironment
TNF-α	Tumor necrosis factor α
IL	Interleukin
IGF-1	Insulin-like growth factor 1
HIF1	Hypoxia-inducing factor
VEGF	Vascular endothelial growth factor
CAF	Cancer-associated fibroblast
TAM	Tumor-associated macrophage
NSCLC	Non-small cell lung cancer
RCC	Renal cell carcinoma
HCC	Hepatocellular cancer
PD-1	Programmed death 1
TMB	Tumor mutational burden
MSI	Microsatellite instability
OS	Overall survival
PFS	Progression-free survival
HR	Hazard ratio
SFI	Subcutaneous fat index
VFI	Visceral fat index
SMI	Skeletal muscle index
NLR	Neutrophil-to-lymphocyte ratio
HER	Electronic health record
MAPKi	Mitogen-activated protein kinase inhibitor
SII	Systemic immune-inflammation index
ORR	Objective response rate
SFM	Subcutaneous fat mass
FBM	Fat body mass
IMDC	International Metastatic RCC Database Consortium
UC	Urothelial cancer
EC	Endometrial cancer
VAT	Visceral adipose tissue
irAEs	Immune-related adverse events
DCR	Disease control rate
dMMR	Deficient mismatch repair
CRC	Colorectal cancer
MSI-H	Microsatellite instability high
DIO	Diet-induced obesity
CD	Control diet
HFD	High-fat diet
IFN	Interferon
HNSCC	Head and neck squamous cell carcinoma
